# Novel metabolic indices and incident type 2 diabetes among women and men: the Rotterdam Study

**DOI:** 10.1007/s00125-019-4921-2

**Published:** 2019-06-10

**Authors:** Adela Brahimaj, Fernando Rivadeneira, Taulant Muka, Eric J. G. Sijbrands, Oscar H. Franco, Abbas Dehghan, Maryam Kavousi

**Affiliations:** 1 Department of Epidemiology, Erasmus University Medical Center, Rotterdam, the Netherlands; 2 Department of General Practice, Erasmus University Medical Center, P. O. Box 2040, 3000 CA Rotterdam, the Netherlands; 3 Department of Internal Medicine, Erasmus University Medical Center, Rotterdam, the Netherlands; 4 Institute of Social and Preventive Medicine (ISPM), University of Bern, Bern, Switzerland; 5 Department of Biostatistics and Epidemiology, MRC-PHE Centre for Environment and Health, School of Public Health, Imperial College London, London, UK

**Keywords:** Android fat, BMI, Combined indices, DXA, Epidemiology, Gynoid fat, LAP, TyG, Type 2 diabetes, VAI

## Abstract

**Aims/hypothesis:**

Both visceral and truncal fat have been associated with metabolic disturbances. We aimed to investigate the associations of several novel metabolic indices, combining anthropometric and lipid measures, and dual-energy x-ray absorptiometry (DXA) measurements of body fat, with incident type 2 diabetes among women and men from the large population-based Rotterdam Study.

**Methods:**

Cox proportional hazards models were used to investigate associations of visceral adiposity index (VAI), lipid accumulation product (LAP), the product of triacylglycerol and glucose (TyG), their formula components and DXA measures with incident type 2 diabetes. Associations were adjusted for traditional diabetes risk factors.

**Results:**

Among 5576 women and 3988 men free of diabetes, 511 women and 388 men developed type 2 diabetes during a median follow-up of 6.5 years. In adjusted models, the three metabolic indices VAI (per 1 SD naturally log-transformed HR; 95% CI) (1.49; 1.36, 1.65 in women; 1.37; 1.22, 1.53 in men), LAP (1.35; 1.16, 1.56 in women; 1.19; 1.01, 1.42 in men) and TyG (1.73; 1.52, 1.98 in women; 1.43; 1.26, 1.62 in men), gynoid fat mass (0.63; 0.45, 0.89) and android to gynoid fat ratio (1.51; 1.16, 1.97) in women were associated with incident type 2 diabetes. BMI (1.45; 1.28, 1.65) was the strongest predictor of type 2 diabetes in men.

**Conclusions/interpretation:**

Among women, novel combined metabolic indices were stronger risk markers for type 2 diabetes than the traditional anthropometric and laboratory measures and were comparable with DXA measures. Neither combined metabolic indices nor DXA measures were superior to traditional anthropometric and lipid measures in association with type 2 diabetes among men.

**Electronic supplementary material:**

The online version of this article (10.1007/s00125-019-4921-2) contains peer-reviewed but unedited supplementary material, which is available to authorised users.

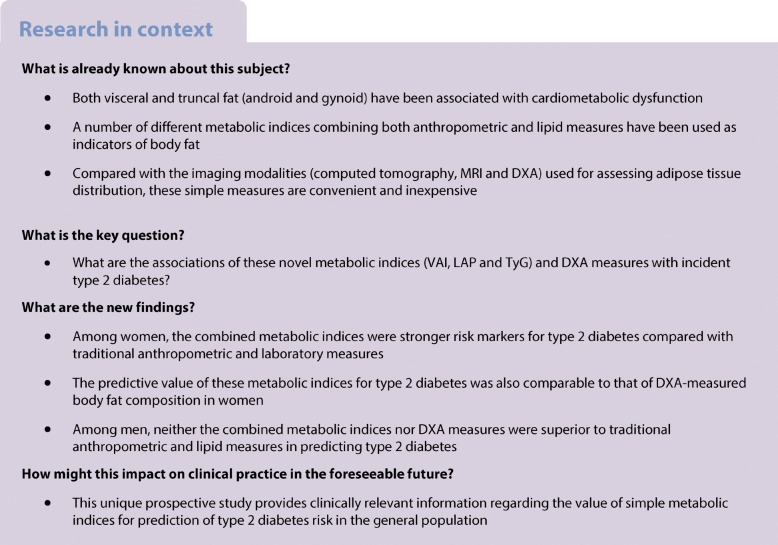



## Introduction

The location of fat accumulation in the body, rather than total fat volume, is increasingly shown to be more important for the risk of type 2 diabetes [1]. Both visceral adipose tissue (VAT) and truncal fat depot have been associated with type 2 diabetes [2–4] and the metabolic syndrome [5, 6].

VAT is a hormonally active component of body fat. The risk of developing diabetes has been shown to be higher in individuals with excess visceral adiposity, with [3] or without [7] manifestations of obesity. Therefore, VAT plays a key role in the association between adiposity and glucose metabolism [4, 8–10]. However, traditional anthropometric measures such as BMI and waist circumference (WC) are not able to distinguish VAT from subcutaneous adipose tissue [11]. Furthermore, VAT accounts for an increased cardiometabolic risk regardless of BMI levels [12]. Truncal fat depot can be partitioned into upper body (android or central) and lower body (gynoid or peripheral) areas. High android to gynoid per cent fat ratio has shown a greater correlation with cardiometabolic dysregulation compared with BMI [13]. Among the elderly, the android fat depot seems to be more closely associated with the metabolic syndrome compared with abdominal visceral fat [5].

Computed tomography (CT) [2, 12] and MRI [3] are the gold standard measures for quantification of VAT. Dual-energy x-ray absorptiometry (DXA) is a well-validated imaging method for precise measurement of body fat mass in various body compartments (i.e. android and gynoid fat) [14]. However, these imaging modalities for assessing adipose tissue distribution are inconvenient and expensive. Recently, different metabolic indices combining both anthropometric and lipid measures have been used as estimators of visceral adiposity dysfunction [15] and lipid overaccumulation [16, 17]. These novel indices, including visceral adiposity index (VAI), lipid accumulation product (LAP) and the product of triacylglycerol (TG) and glucose (TyG), have been suggested as early markers of insulin resistance, mainly in cross-sectional studies [18–20]. However, the associations of these novel metabolic indices with incident type 2 diabetes remain unclear. We therefore studied the associations of different novel metabolic indices and their formula components with incident type 2 diabetes among women and men from the large prospective population-based Rotterdam Study. We further assessed the associations of truncal fat depot measured by DXA with incident type 2 diabetes.

## Methods

### Study population

The study was performed in the framework of the Rotterdam Study, a prospective population-based cohort study carried out in Ommoord, a district of Rotterdam, the Netherlands. The design of the Rotterdam Study has been described in more detail elsewhere [21]. The original cohort (RSI) started in 1989 when all residents within the well-defined study area aged 55 years or older were invited to participate, of whom 78% (7983 out of 10,275) accepted. The first examination of the original cohort (RSI-1) took place from 1990 to 1993. The cohort has been extended twice (RSII in 2000 and RSIII in 2006) to include participants who were 45 years or older or who had moved to the study research area. For all three cohorts of the Rotterdam Study, follow-up examinations were conducted every 3–5 years. The study was approved according to the Population Screening Act: Rotterdam Study by the medical ethics committee of the Netherlands Ministry of Health, Welfare and Sport. All participants provided written informed consent to take part in the study and allow investigators to obtain information from their treating physicians.

The current study was based on data collected during the third visit of the first cohort (RSI-3; 1997-1999), the first visit of the second cohort (RSII-1; 2000-2001) and the first visit of the third cohort (RSIII-1; 2006-2008). From 11,740 individuals in the three visits, diabetes data were available for 10,898 (6241 women and 4657 men). After excluding 1334 prevalent diabetes cases (665 women and 669 men), 9564 people (5576 women and 3988 men) were included in the analyses of different metabolic indices and incident type 2 diabetes. DXA body fat measurements were available for 3518 individuals (2026 women and 1492 men) with available diabetes data at the fourth visit of the first cohort (RSI-4; 2002-2004) and the second visit of the second cohort (RSII-2; 2004-2005). After excluding 556 prevalent diabetes cases (292 women and 264 men) at the time of DXA measurement, 2962 individuals (1734 women and 1228 men) were included in the analyses of DXA measures of body fat and incident type 2 diabetes.

### Combined metabolic indices

Novel metabolic indices combine anthropometric measures such as BMI and WC with lipid measures: TG, HDL-cholesterol or fasting plasma glucose (FPG).

LAP, VAI and TyG were calculated using published formulae. LAP was calculated as LAP = (WC − 65) × TG for men and LAP = (WC − 58) × TG for women [22]. VAI was calculated as VAI $=\left[\left(\frac{\mathrm{WC}}{39.68}\right)+\left(1.88\times \mathrm{BMI}\right)\right]\times \left(\frac{\mathrm{TG}}{1.03}\right)\times \left(\frac{1.31}{\mathrm{HDL}}\right)$ for men and VAI $=\left[\left(\frac{\mathrm{WC}}{36.58}\right)+\left(1.89\times \mathrm{BMI}\right)\right]\times \left(\frac{\mathrm{TG}}{0.81}\right)\times \left(\frac{1.52}{\mathrm{HDL}}\right)$ for women [15]. In both formulae, TG and HDL-cholesterol levels are expressed in mmol/l, WC in cm and BMI in kg/m^2^. The TyG index was calculated as ${\mathrm{Log}}_e\left(\mathrm{TG}\times \frac{\mathrm{FPG}}{2}\right)$, where both TG and FPG are expressed in mg/dl [18, 20].

### DXA measurements of body fat

Body composition was assessed using DXA. A Prodigy total body-fat beam densitometer (GE Lunar, Madison, WI, USA) was used to perform whole-body DXA scans [21]. Total body weight (g) was divided into bone mineral content, lean mass and fat mass. In addition, fat mass of the android and gynoid body regions was analysed. Total per cent fat mass, per cent android fat and per cent gynoid fat were calculated as percentages of total body weight. The ratio of per cent android to gynoid fat mass was also calculated.

### Ascertainment of type 2 diabetes mellitus

Participants were followed from the date of their baseline visit onwards. Cases of type 2 diabetes were ascertained through active follow-up using general practitioners’ records, hospital discharge letters, pharmacy data and glucose measurements from study visits which took place approximately every 4 years [23]. In the Rotterdam Study, type 2 diabetes ascertainment was done in the same way for all individuals, avoiding substantial potential for misclassification or ascertainment bias. According to the current WHO guidelines, type 2 diabetes was defined as FPG ≥7.0 mmol/l, non-FPG ≥11.1 mmol/l (when fasting samples were unavailable) or use of blood glucose-lowering medication [24]. Information regarding the use of blood glucose-lowering medication was derived from both structured home interviews and linkage to pharmacy dispensing records. At baseline, more than 95% of the Rotterdam Study population was covered by the pharmacies in the study area. All potential events of type 2 diabetes were independently adjudicated by two study physicians. In case of disagreement, consensus was sought with an endocrinologist. Follow-up data were complete until 1 January 2012 [25]. 

### Assessment of covariates

Height and weight were measured with the participants standing without shoes and heavy outer garments. Waist circumference was measured at the level midway between the lower rib margin and the iliac crest with participants in a standing position without heavy outer garments and with emptied pockets, breathing out gently. Blood pressure was measured twice at the right brachial artery with a random-zero sphygmomanometer with the participant in a sitting position, and the mean of two consecutive measurements was used. Insulin, glucose, HDL-cholesterol, and TG were measured on the COBAS 8000 Modular Analyzer (Roche Diagnostics GmbH). HOMA-IR and HOMA-B were calculated as described previously [26]. TG levels were not available at the same visit as DXA measures (RSI-4) and thus they were taken from the closest previous visit (RSI-3). The corresponding interassay coefficients of variation are the following: insulin <8%, glucose <1.4% and lipids <2.1%. Information on medication use and tobacco smoking behaviour was collected by trained research assistants via computerised questionnaires during home visits. Smoking was classified as current vs non-current smokers. History of cardiovascular disease (CVD) was defined as a history of coronary heart disease (myocardial infarction, revascularisation, coronary artery bypass graft surgery or percutaneous coronary intervention) or stroke and was verified from the medical records of the general practitioners.

### Statistical analysis

Considering sex differences in fat distribution and that the formulae of metabolic indices differ by sex, all analyses were performed among women and men separately. Descriptive characteristics were presented as means ± SD for continuous variables, and numbers (percentages) for dichotomous variables. One-way ANOVA for continuous variables and the χ^2^ test for categorical variables were used to compare general characteristics between women and men as well as between participants with and without DXA measurements. Markers with a right-skewed distribution (insulin, glucose, HDL-cholesterol, TG, VAI, LAP, per cent android fat, per cent gynoid fat, android to gynoid fat ratio and per cent total fat mass) were transformed to the natural logarithmic scale.

Cox proportional hazards models were used to investigate associations of different combined metabolic indices (VAI, LAP, TyG), the anthropometric (BMI, WC) or laboratory components (inverse HDL-cholesterol, TG) included in their formulae, as well as DXA measurements of body fat (android, gynoid, total fat mass, the ratio of per cent android to gynoid fat mass) with incident type 2 diabetes. Inverse HDL-cholesterol was used to facilitate easier comparison between the estimates. The proportional hazards assumption of the Cox model was checked by visual inspection of log minus log plots and by performing a test for heterogeneity of the exposure over time. There was no evidence of violation of the proportionality assumption in any of the models (*p* value for time-dependent interaction terms >0.05). The first model was adjusted for age and cohort. The second model was additionally adjusted for BMI. The third model was additionally adjusted for systolic BP, hypertension medication, smoking and prevalent CVD. The fourth model was additionally adjusted for HDL-cholesterol, TG and serum lipid-reducing agents. In the fifth model, FPG was added. As glucose measurement is a means to diagnose type 2 diabetes, model 5 should be considered a conservative model. For each novel lipid index, the covariates that were already in the index formula were excluded from the multivariable-adjusted model.

To check whether the association of different markers with incident diabetes differed by obesity status, the analyses were further stratified based on a BMI cut-off of 30 kg/m^2^ and performed among non-obese (BMI <30 kg/m^2^) and obese (BMI ≥30 kg/m^2^) individuals. The *p* value is derived from the *z* score calculated from the ratio between the difference of the two estimates and the SE of the difference [27]. The *p* value indicates whether the difference between the estimates is significant. To compare the estimates between women and men, an interaction test was applied to model 4 (analyses of the total population).

Multiple imputation procedure was performed (five imputations) to impute missing data for covariates. All analyses were conducted using IBM SPSS software, version 21 (IBM, Armonk, NY, USA). A *p* value below 0.05 was considered statistically significant.

## Results

### Metabolic indices and incident type 2 diabetes

Baseline characteristics of 5576 women and 3988 men included in the study are shown in Table 1. Women were older, had lower levels of systolic BP and glucose but higher levels of total cholesterol. A larger proportion of women were treated for hypertension. CVD was more prevalent among men, and a larger proportion of men were receiving lipid-reducing agents or were current smokers. BMI, HDL-cholesterol, TG and VAI were higher in women, whereas WC, LAP and TyG were higher in men (Table 1).

**Table 1:** Baseline characteristics of study participants (*N* = 9564)

Characteristic	Women (*n*=5576)	Men (*n*=3988)	*p* value
Age, years	65.1 ± 10.3	64.3 ± 9.5	<0.001
Systolic BP, mmHg	136.2 ± 21.6	138.6 ± 20.2	<0.001
Treatment for hypertension	1225 (22.0)	786 (19.7)	0.011
Prevalent CVD	282 (5.1)	564 (14.1)	<0.001
Serum lipid-reducing agent use	739 (13.3)	639 (16.0)	0.001
Current smoker	809 (14.5)	874 (21.9)	<0.001
Total cholesterol, mmol/l	5.9 ± 0.9	5.5 ± 0.9	<0.001
Insulin, pmol/l	69.0 (30.0–182.0)	71.0 (30.0–188.0)	0.2
Glucose, mmol/l	5.3 (4.6–6.4)	5.5 (4.7–6.5)	<0.001
Metabolic indices			
BMI, kg/m^2^	27.1 ± 4.5	26.7 ± 3.4	<0.001
WC, cm	89.1 ± 11.8	97.7 ± 10.0	<0.001
HDL-cholesterol, mmol/l	1.5 (0.9–2.3)	1.2 (0.8–1.9)	<0.001
TG, mmol/l	1.3 (0.7–2.8)	1.3 (0.7–3.1)	<0.001
VAI	1.6 (0.6–4.8)	1.5 (0.6–4.8)	0.008
LAP	38.1 (11.4–106.8)	42.6 (15.7–122.4)	<0.001
TyG	2.8 ± 0.5	2.9 ± 0.5	<0.001
DXA measurements^a^			
Android fat, %	3.3 (1.8–4.5)	3.1 (1.6–4.3)	<0.001
Gynoid fat, %	6.3 (4.5–8.1)	3.9 (2.6–5.3)	<0.001
Android to gynoid fat ratio, %	0.5 (0.3–0.7)	0.8 (0.5–1.1)	<0.001
Total fat mass, %	39.3 (27.2–48.6)	27.6 (16.9–37.1)	<0.001

Values are presented as means ± SD, median (interquartile range) or *n* (%)

^a^Of the 9564 participants, DXA measurements were available for 1770 women and 1258 men (*n*=3028). The baseline characteristics of participants with DXA measurements differed significantly (*p*<0.001) from those of participants without DXA measurements but were not significantly different for prevalent CVD (*p*=0.3), HDL-cholesterol (*p*=0.055) and TG (*p*=0.7). However, given that they were in the same cohorts of the Rotterdam Study, but had different visits, participants with DXA measurements included in the analyses are a subset of the study sample without DXA measurements, who survived until the next Rotterdam Study visit, when DXA was measured

*p* values are for the comparison of baseline characteristics between women and men

The correlation coefficients for metabolic indices in relation to glycaemic indices are shown in ESM Table 1. For both women and men, the correlation coefficients for VAI, LAP and TyG ranged between 0.43 and 0.57 for HOMA-IR and between 0.04 and 0.28 for HOMA-B. The correlation coefficients for different visceral fat indices in relation to HOMA-IR were overall larger among women compared with men, albeit not statistically significantly.

During a median follow-up of 6.5 years (maximum 14.7 years) 899 cases of incident type 2 diabetes were identified (511 women and 388 men). All indices were significantly associated with the risk of type 2 diabetes in age-adjusted models (model 1). In the multivariable-adjusted model (model 4), TyG showed the largest association with type 2 diabetes in both women (per 1 SD HR; 95% CI) (1.73; 1.52, 1.98) and in men (1.43; 1.26, 1.62). Other markers that remained significantly associated with incident type 2 diabetes in both sexes in the multivariable-adjusted model were BMI (1.37; 1.26, 1.49 in women and 1.45; 1.28, 1.65 in men), inverse HDL-cholesterol (per 1 SD naturally log-transformed HR; 95% CI) (1.29; 1.14, 1.46 in women and 1.32; 1.14, 1.52 in men), VAI (1.49; 1.36, 1.65 in women and 1.37; 1.22, 1.53 in men) and LAP (1.35; 1.16, 1.56 in women and 1.19; 1.01, 1.42 in men). WC (1.24; 1.07, 1.45) and TG (1.24; 1.10, 1.39) remained strongly associated with the risk of type 2 diabetes only in women (Table 2). Associations of metabolic indices with diabetes were overall larger among women compared with men. However, the difference of the estimates between women and men was statistically significant only for TyG (Table 2).

**Table 2:** Associations between different metabolic indices and incident type 2 diabetes (*N* = 9564)

Index	Incident type 2 diabetes HR (95% CI)	
	Women (511 cases)	Men (388 cases)
BMI		
Model 1	^*^1.51 (1.39, 1.63)	^*^1.64 (1.45, 1.86)
Model 2	NA	NA
Model 3	^*^1.49 (1.38, 1.62)	^*^1.61 (1.42, 1.82)
Model 4	^*^1.37 (1.26, 1.49)	^*^1.45 (1.28, 1.65)
Model 5	^*^1.27 (1.17, 1.38)	^*^1.25 (1.09, 1.43)
WC		
Model 1	^*^1.62 (1.49, 1.77)	^*^1.44 (1.31, 1.58)
Model 2	^*^1.39 (1.19, 1.61)	1.15 (0.94, 1.39)
Model 3	^*^1.37 (1.18, 1.59)	1.13 (0.92, 1.38)
Model 4	^*^1.24 (1.07, 1.45)	1.04 (0.83, 1.31)
Model 5	1.04 (0.89, 1.22)	1.04 (0.82, 1.30)
1/HDL^a^		
Model 1	^*^1.58 (1.44, 1.74)	^*^1.53 (1.36, 1.73)
Model 2	^*^1.46 (1.33, 1.61)	^*^1.42 (1.25, 1.61)
Model 3	^*^1.46 (1.32, 1.61)	^*^1.40 (1.24, 1.59)
Model 4	^*^1.29 (1.14, 1.46)	^*^1.32 (1.14, 1.52)
Model 5	^*^1.29 (1.14, 1.47)	^*^1.41 (1.22, 1.63)
TG^a^		
Model 1	^*^1.58 (1.44, 1.74)	^*^1.44 (1.30, 1.58)
Model 2	^*^1.45 (1.31, 1.60)	^*^1.30 (1.18, 1.45)
Model 3	^*^1.41 (1.28, 1.56)	^*^1.28 (1.15, 1.42)
Model 4	^*^1.24 (1.10, 1.39)	1.12 (0.99, 1.27)
Model 5	1.07 (0.95, 1.21)	0.94 (0.83, 1.06)
VAI^a^		
Model 1	^*^1.65 (1.51, 1.81)	^*^1.52 (1.36, 1.69)
Model 2	NA	NA
Model 3	^*^1.49 (1.35, 1.65)	^*^1.37 (1.22, 1.53)
Model 4	^*^1.49 (1.36, 1.65)	^*^1.37 (1.22, 1.53)
Model 5	^*^1.29 (1.17, 1.43)	^*^1.23 (1.09, 1.38)
LAP^a^		
Model 1	^*^1.83 (1.65, 2.03)	^*^1.66 (1.47, 1.87)
Model 2	^*^1.60 (1.41, 1.82)	^*^1.47 (1.27, 1.70)
Model 3	^*^1.55 (1.36, 1.76)	^*^1.43 (1.24, 1.66)
Model 4	^*^1.35 (1.16, 1.56)	^*^1.19 (1.01, 1.42)
Model 5	1.08 (0.93, 1.26)	0.96 (0.81, 1.15)
TyG		
Model 1	^*^2.06 (1.86, 2.29)	^*^1.74 (1.56, 1.94)
Model 2	^*^1.88 (1.69, 2.09)	^*^1.58 (1.41, 1.77)
Model 3	^*^1.82 (1.64, 2.04)	^*^1.55 (1.38, 1.75)
Model 4^b^	^*^1.73 (1.52, 1.98)	^*^1.43 (1.26, 1.62)
Model 5	NA	NA

HRs are presented per 1 SD increase in the marker

Model 1, adjusted for age and cohort; model 2, additionally adjusted for BMI; model 3, additionally adjusted for systolic BP, treatment for hypertension, smoking and prevalent CVD; model 4, additionally adjusted for HDL-cholesterol, TG and serum lipid-reducing agents; model 5, additionally adjusted for FPG

^a^Marker is log_*e*_ transformed

^b^*p* value from the interaction test for the difference in HR between women and men <0.05

*p<0.05, by Cox proportional hazards model

NA, not applicable

After additionally adjusting for FPG (model 5), only BMI (1.27; 1.17, 1.38 for women and 1.25; 1.09, 1.43 for men), inverse HDL-cholesterol (1.29; 1.14, 1.47 for women and 1.41; 1.22, 1.63 for men) and VAI (1.29; 1.17, 1.43 for women and 1.23; 1.09, 1.38 for men) remained significantly associated with the risk of type 2 diabetes in both sexes (Table 2).

In the analyses stratified for obesity status, in the multivariable-adjusted model (model 4), BMI, inverse HDL-cholesterol, VAI and TyG remained significantly associated with incident diabetes, regardless of obesity status. While LAP was significantly associated with incident diabetes among non-obese women and men, WC and TG remained strongly associated with the risk of type 2 diabetes only in non-obese women. Overall, the tendency for the associations of visceral fat indices with diabetes was stronger among non-obese individuals (ESM Table 2).

### DXA measurements of body fat and incident type 2 diabetes

Android fat, gynoid fat and per cent total fat mass were higher in women, whereas the ratio of per cent android to gynoid fat was higher in men (Table 1). Complete baseline characteristics of 1770 women and 1258 men included in the analyses of DXA measurements and type 2 diabetes are presented in ESM Table 3.

Among 1770 women and 1258 men in the DXA measurement analyses, 185 women and 137 men developed type 2 diabetes during a median follow-up of 8 years (maximum 10 years). Per cent gynoid fat (per 1 SD naturally log-transformed HR; 95% CI) (0.63; 0.45, 0.89) and the ratio of per cent android to gynoid fat (1.51; 1.16, 1.97) remained significantly associated with incident type 2 diabetes in the multivariable-adjusted model (model 4) only in women (Table 3).

**Table 3:** Associations between DXA measurements of body fat and incident type 2 diabetes (*N* = 3028)

DXA measurement	Incident type 2 diabetes HR (95% CI)	
	Women (185 cases)	Men (137 cases)
Android fat mass, %^a^		
Model 1	^*^1.77 (1.42, 2.22)	^*^1.43 (1.13, 1.81)
Model 2	^*^1.42 (1.06, 1.89)	^*^1.44 (1.06, 1.95)
Model 3	^*^1.36 (1.02, 1.82)	^*^1.41 (1.04, 1.92)
Model 4	1.22 (0.91, 1.64)	1.32 (0.96, 1.83)
Model 5	1.10 (0.83, 1.46)	1.33 (0.96, 1.85)
Gynoid fat mass, %^a^		
Model 1	1.01 (0.76, 1.35)	1.21 (0.91, 1.59)
Model 2	^*^0.56 (0.40, 0.78)	1.03 (0.74, 1.44)
Model 3	^*^0.57 (0.41, 0.79)	1.03 (0.74, 1.44)
Model 4^b^	^*^0.63 (0.45, 0.89)	1.12 (0.78, 1.59)
Model 5	0.76 (0.54, 1.07)	1.08 (0.76, 1.55)
Android to gynoid fat ratio^a^		
Model 1	^*^1.95 (1.55, 2.46)	^*^1.56 (1.16, 2.11)
Model 2	^*^1.73 (1.36, 2.22)	^*^1.49 (1.09, 2.04)
Model 3	^*^1.69 (1.32, 2.17)	^*^1.46 (1.06, 1.99)
Model 4	^*^1.51 (1.16, 1.97)	1.26 (0.91, 1.76)
Model 5	1.28 (0.98, 1.67)	1.32 (0.93, 1.88)
Total fat mass, %^a^		
Model 1	^*^1.56 (1.17, 2.08)	^*^1.43 (1.11, 1.84)
Model 2	0.77 (0.53, 1.11)	^*^1.43 (1.00, 2.04)
Model 3	0.75 (0.52, 1.08)	1.41 (0.98, 2.02)
Model 4^b^	0.76 (0.52, 1.13)	1.45 (0.99, 2.12)
Model 5	0.86 (0.59, 1.26)	1.45 (0.99, 2.12)

HRs are presented per 1 SD increase in the marker

Model 1, adjusted for age and cohort; model 2, additionally adjusted for BMI; model 3, additionally adjusted for systolic BP, treatment for hypertension, smoking and prevalent CVD; model 4, additionally adjusted for HDL-cholesterol, TG and serum lipid-reducing agents; model 5, additionally adjusted for FPG

^a^Marker is log_*e*_ transformed

^b^*p* value from the interaction test for the difference in HR between women and men <0.05

*p<0.05, by Cox proportional hazards model

In the analyses stratified for obesity status, per cent gynoid fat (0.57; 0.38, 0.84) and the ratio of per cent android to gynoid fat (1.77; 1.29, 2.41) remained significantly associated with incident type 2 diabetes in the multivariable-adjusted model (model 4) only in non-obese women (ESM Table 4). After additionally adjusting for FPG (model 5), only the ratio of per cent android to gynoid fat mass (1.51; 1.09, 2.08) remained associated with incident type 2 diabetes in non-obese women (ESM Table 4).

## Discussion

In the large population-based Rotterdam Study, the novel metabolic indices VAI, LAP and TyG were stronger risk markers for incident diabetes compared with traditional anthropometric and lipid measures among women. The magnitude of association of these novel metabolic indices with diabetes was comparable with that of DXA-measured body fat compositions in women. Among men, neither combined metabolic indices nor DXA measures of body fat were superior to traditional anthropometric and lipid measures, in particular BMI, in association with diabetes.

VAT is a hormonally active component of total body fat, which may play a key role in the association between adiposity and glucose metabolism [4, 8–10]. Excess visceral adiposity has been linked to a higher risk of type 2 diabetes, regardless of obesity [2, 3, 7, 12]. The three combined metabolic indices VAI, LAP and TyG have been introduced as indicators of ‘visceral adipose function’ [15] and insulin resistance [18–20] and have been linked to cardiometabolic risk [15], prediabetes [28] and diabetes [28] in cross-sectional studies. Our study is the first to simultaneously investigate the longitudinal associations of all these new indices, as well as their components, with incident type 2 diabetes among women and men. The three novel combined metabolic indices were all independently associated with increased risk of diabetes in our study. VAI and LAP combine both anthropometric and metabolic variables to evaluate, respectively, adiposity dysfunction and lipid overaccumulation, whereas TyG includes only metabolic variables. TyG is among the most mentioned insulin resistance indices in the literature [29–36]. TyG has also been suggested as a promising biomarker for glycaemic control in people with type 2 diabetes [30], even better than HOMA [29]. In comparison with FPG, TyG improved diabetes risk prediction in individuals with normal FPG [37]. LAP includes WC and TG, similarly to hypertriglyceridaemic waist [17], and is an index of excessive lipid accumulation. Since precise measurement of visceral fat content requires the use of expensive imaging techniques such as CT or MRI [2, 12], simple and economical quantification of these visceral adiposity indices could lead to improvements in identification of individuals at high risk of developing type 2 diabetes.

The counterbalance between insulin secretion and insulin resistance is critical for type 2 diabetes pathogenesis. VAI, LAP and TyG have been introduced as early indicators of insulin resistance [18–20]. In our study, these three indices were all moderately correlated with an index of insulin resistance (HOMA-IR) and showed a smaller correlation with insulin secretion (HOMA-B). As VAI and LAP combine both lipid variables and adiposity status, they could serve as better surrogates for insulin resistance compared with either lipid or adiposity measures alone. The largest correlation of TyG with insulin resistance in our study is in line with other study findings, supporting a central role of both lipotoxicity and glucotoxicity in modulating insulin resistance [38]. Since obesity has a strong impact on dyslipidaemia, insulin resistance and the development of type 2 diabetes, we further stratified the analyses based on obesity status. Correlation of different combined adiposity indices with HOMA measures did not materially differ between non-obese and obese individuals. The overall tendency towards stronger associations of these metabolic indices with incident diabetes among non-obese individuals might be due to their lower discriminatory power among higher risk obese individuals.

While the exact mechanisms responsible for the relationship between excess abdominal/visceral fat and cardiometabolic risk are still unclear, several hypotheses have been proposed [39–41]. Subcutaneous fat faces obesogenic stress with a limited capacity for regional adipocyte hypertrophy or hyperplasia. Once this capacity is surpassed, adipose tissue storage is forced into other regions, such as organs or compartments of the body, termed ectopic. Visceral fat is considered the classic ectopic fat depot and is associated with dysfunctional adiposity or adiposopathy [42, 43].

In our study, WC, TG, VAI, LAP and TyG showed a stronger association with incident type 2 diabetes among women compared with men. Similarly, the correlations between VAI, LAP and TyG with HOMA-IR in our study were overall stronger among women. The greater association of VAT with diabetes and adverse cardiovascular risk profiles among women has been suggested in several studies [44, 45]. Sex differences in adverse metabolic outcomes associated with visceral fat have been related to a significantly lower visceral fat area in non-diabetic women compared with non-diabetic men, and a similar visceral fat area for both diabetic women and men [44]. Among individuals with more visceral fat, a greater portion of hepatic NEFA delivery originates from VAT lipolysis [46]. Contribution of visceral lipolysis to hepatic NEFA delivery in relation to visceral fat has been found to be greater in women than in men [46]. Moreover, correlation between VAT area and serum TG has been found to be stronger in women than in men [47].

No previous study has investigated the associations of DXA measures of body fat with incident type 2 diabetes. Our study suggests that per cent gynoid fat and per cent android to gynoid fat ratio among women and total fat mass among men are independent risk markers for diabetes. Previous studies have shown important relations between android to gynoid fat ratio and metabolic risk in healthy adults. Android or truncal obesity has been associated with the risk of metabolic disorders and CVD [48], yet there is evidence that gynoid fat distribution may be protective [49]. Android fat depot is the adipose tissue mainly around the trunk including, but not exclusively, visceral fat. Compared with abdominal visceral fat, android fat depot has shown a larger association with the metabolic syndrome in elderly people [5]. In line with our findings, high per cent android to gynoid fat ratio has shown a larger correlation with cardiometabolic dysregulation compared with per cent android fat, per cent gynoid fat or BMI [13]. Compared with women with a predominantly gynoid fat distribution, android obesity in women has been correlated with a higher incidence of glucose intolerance [50]. Excess android fat mass has recently been associated with high TG and low HDL-cholesterol levels in men and high LDL- and low HDL-cholesterol levels in women. Excess gynoid fat mass has been positively correlated with total cholesterol in men and has shown a favourable association with TG and HDL-cholesterol in women [51]. Increased gynoid fat mass has also been shown to be protective against the progression of non-alcoholic fatty liver disease in Japanese women with type 2 diabetes [52]. It therefore seems that regional fat distribution in the android and gynoid regions have varying effects on lipid profiles among women and men. In line with this, we found an inverse association in women between gynoid fat and android to gynoid fat ratio and type 2 diabetes and a positive association in men between total fat mass and type 2 diabetes.

In our study, the magnitude of association between DXA measures of body fat and diabetes was comparable with that of combined metabolic indices and traditional anthropometric and lipid measures. Considering the costs and radiation exposure associated with DXA measurement, its use in the general population as a screening tool for diabetes may therefore not be justified, and using well-established and simple anthropometric variables such as BMI might suffice.

To our knowledge, this is the first prospective population-based cohort study to simultaneously investigate the associations between novel metabolic indices as well as DXA measures with incident diabetes among women and men over a long follow-up period. We used data from a well-characterised prospective cohort study, which allowed for direct comparison of several metabolic indices as well as correction for a wide range of covariates.

The limitations of our study also warrant attention. Our population comprised individuals aged 45 years and older of European ancestry. One might speculate that the impact of VAT on diabetes incidence would have been even stronger in a younger population. Thus, generalisation of our results to younger age groups and other ethnicities should be made with caution. Moreover, as with other cohort studies, the possibility of selection bias could not be entirely ruled out. Due to the unavailability of CT or MRI in our population, visceral adiposity was not directly measured but estimated. Also, we did not have DXA measures specifically for visceral fat in the Rotterdam Study. Instead, android fat measured by DXA was used as a proxy for visceral fat. Thus, comparison of our results against the gold standard measures for visceral fat is not possible. We did not include variables such as socioeconomic status, family history of diabetes, dietary intake and physical activity in our multivariable models, as they were not available.

In conclusion, the novel combined metabolic indices VAI, LAP and TyG were stronger risk markers for incident type 2 diabetes compared with traditional anthropometric and lipid measures among women. The predictive value of these novel metabolic indices for type 2 diabetes was also comparable with that of DXA-measured body fat compositions in women. Neither combined metabolic indices nor DXA measures of body fat were superior to traditional anthropometric and lipid measures in association with type 2 diabetes among men. In particular, BMI remained the best marker for type 2 diabetes risk in men and among the best markers in women. BMI could therefore be used as a simple and useful tool for diabetes risk screening in the general population.

## Electronic supplementary material

ESM Tables (PDF 92 kb)

## Data Availability

The datasets generated during and/or analysed during the current study are available from the corresponding author on reasonable request.

## Abbreviations

CT: Computed tomography
CVD: Cardiovascular disease
DXA: Dual-energy x-ray absorptiometry
FPG: Fasting plasma glucose
LAP: Lipid accumulation product
TG: Triacylglycerols
TyG: Product of triacylglycerol and glucose
VAI: Visceral adiposity index
VAT: Visceral adipose tissue
WC: Waist circumference
