# Supplementary material for: Novel metabolic indices and incident type 2 diabetes among women and men: the Rotterdam Study
Source: Diabetologia. 2019 Jun 10;62(9):1581–90. doi: 10.1007/s00125-019-4921-2 (PMC6677703; doi:10.1007/s00125-019-4921-2)
Supplement: Supplementary file 1 — (PDF 92 kb) [file 125_2019_4921_MOESM1_ESM.pdf]

Electronic supplement material (ESM):

ESM Table 1. Correlation of metabolic indices and DXA measures with glycemic indices.

ESM Table 2. Associations between different metabolic indices and incident type 2 diabetes mellitus in obese/non-obese subjects.

ESM Table 3. Baseline characteristics of study participants with DXA measurements on body fat.

ESM Table 4. Associations between DXA measurements on body fat and incident type 2 diabetes in obese/non-obese subjects.

ESM Table 1.Correlation of metabolic indices and DXA measures with glycemic indices.

	Pearson`s Correlation Coefficient				
Sex		FG	FI	HOMA-IR	HOMA-B
Women	BMI	0.270*	0.491*	0.494*	0.287*
	WC	0.295*	0.501*	0.510*	0.271*
	1/HDL	0.210*	0.352*	0.358*	0.198*
	Triglycerides	0.291*	0.398*	0.424*	0.193*
	VAI	0.295*	0.437*	0.455*	0.212*
	LAP	0.341*	0.535*	0.551*	0.273*
	TyG	0.596*	0.483*	0.574*	0.057*
	Android fat mass %	0.201*	NA	NA	NA
	Gynoid fat mass %	-0.001	NA	NA	NA
	Android/Gynoid	0.239*	NA	NA	NA
	Total fat mass %	0.122*	NA	NA	NA
Men	BMI	0.223*	0.485*	0.484*	0.314*
	WC	0.237*	0.478*	0.483*	0.291*
	1/HDL	0.145*	0.319*	0.318*	0.218*
	Triglycerides	0.246*	0.381*	0.399*	0.204*
	VAI	0.240*	0.417*	0.431*	0.242*
	LAP	0.290*	0.506*	0.523*	0.287*
	TyG	0.575*	0.443*	0.547*	0.045*
	Android fat mass %	0.157*	NA	NA	NA
	Gynoid fat mass %	0.116*	NA	NA	NA
	Android/Gynoid	0.098*	NA	NA	NA
	Total fat mass %	0.150*	NA	NA	NA

*Correlation is significant at the 0.01 level (2-tailed).

NA-Not Applicable (insulin was not measured in RSI-4 and RSII-2).

ESM Table 2. Associations between different lipid indices and incident type 2 diabetes mellitus in obese/non-obese subjects (N = 9564).

Index	Incident type 2 diabetes			
	Women		Men	
	BMI < 30 (N=309)	BMI ≥ 30 (N=167)	BMI<30 (N=278)	BMI ≥ 30 (N=79)
	HR(95%CI)	HR(95%CI)	HR(95%CI)	HR(95%CI)
BMI<br>Model 1	*1.79 (1.47, 2.16)	*1.46 (1.22, 1.74)	*1.78 (1.42, 2.24)	*1.61 (1.13, 2.27)
Model 2	NA	NA	NA	NA
Model 3	*1.72 (1.42, 2.09)	*1.47 (1.23, 1.76)	*1.74 (1.55, 1.96)	*1.54 (1.09, 2.18)
Model 4	*1.49 (1.22, 1.82)	*1.42 (1.18, 1.71)	*1.50 (1.18, 1.91)	*1.45 (1.01, 2.06)
Model 5	*1.45 (1.19, 1.77)	*1.24 (1.02, 1.50)	1.23 (0.96, 1.58)	1.09 (0.75, 1.60)
WC<br>Model 1	*1.76 (1.51, 2.05)	*1.38 (1.15, 1.64)	*1.65 (1.37, 1.98)	1.66 (0.91, 1.49)
Model 2	*1.56 (1.28, 1.89)	1.18 (0.93, 1.48)	*1.38 (1.05, 1.83)	0.95 (0.65, 1.39)
Model 3	*1.50 (1.23, 1.83)	1.18 (0.94, 1.49)	*1.35 (1.02, 1.79)	0.91 (0.62, 1.34)
Model 4	*1.35 (1.10, 1.66)	1.06 (0.83, 1.36)	1.26 (0.94, 1.68)	0.84 (0.55, 1.27)
Model 5	1.14 (0.93, 1.41)	0.87 (0.68, 1.13)	1.26 (0.93, 1.71)	0.79 (0.49, 1.29)
[e]1/HDL<br>Model 1	*1.49 (1.34, 1.68)	*1.57 (1.32, 1.87)	*1.47 (1.28, 1.69)	*1.52 (1.18, 1.96)
Model 2	*1.42 (1.26, 1.59)	*1.51 (1.27, 1.81)	*1.39 (1.21, 1.61)	*1.49 (1.15, 1.92)
Model 3	*1.41 (1.25, 1.60)	*1.51 (1.27, 1.81)	*1.38 (1.19, 1.59)	*1.46 (1.12, 1.90)
Model 4	*1.22 (1.05, 1.42)	*1.38 (1.12, 1.71)	*1.28 (1.09, 1.51)	*1.40 (1.04, 1.89)
Model 5	*1.23 (1.05, 1.43)	*1.40 (1.13, 1.74)	*1.39 (1.17, 1.64)	*1.49 (1.09, 2.06)
[e]Triglycerides<br>Model 1	*1.57 (1.39, 1.77)	*1.39 (1.17, 1.65)	*1.41 (1.25, 1.59)	*1.26 (1.03, 1.54)
Model 2	*1.23 (1.05, 1.43)	*1.40 (1.13, 1.74)	*1.39 (1.17, 1.64)	*1.49 (1.09, 2.06)
Model 3	*1.44 (1.28, 1.63)	*1.36 (1.14, 1.62)	*1.30 (1.15, 1.47)	*1.23 (1.00, 1.51)
Model 4	*1.29 (1.12, 1.50)	1.17 (0.95, 1.44)	1.14 (0.99, 1.32)	1.08 (0.86, 1.36)
Model 5	1.10 (0.95, 1.28)	1.03 (0.84, 1.27)	0.98 (0.84, 1.13)	0.90 (0.71, 1.16)
[e]VAI<br>Model 1	*1.62 (1.44, 1.82)	*1.50 (1.27, 1.78)	*1.48 (1.31, 1.69)	*1.44 (1.14, 1.80)
Model 2	NA	NA	NA	NA
Model 3	*1.57 (1.39, 1.77)	*1.49 (1.26, 1.77)	*1.45 (1.27, 1.64)	*1.38 (1.09, 1.75)
Model 4	*1.57 (1.39, 1.77)	*1.50 (1.27, 1.77)	*1.45 (1.27, 1.64)	*1.38 (1.09, 1.75)
Model 5	*1.35 (1.19, 1.52)	*1.34 (1.13, 1.59)	*1.29 (1.14, 1.48)	1.20 (0.94, 1.54)
[e]LAP<br>Model 1	*1.76 (1.54, 2.02)	*1.66 (1.34, 2.07)	*1.65 (1.42, 1.90)	*1.49 (1.12, 1.98)
Model 2	*1.64 (1.41, 1.91)	*1.51 (1.20, 1.91)	*1.53 (1.29, 1.81)	*1.39 (1.03, 1.88)
Model 3	*1.57 (1.35, 1.84)	*1.49 (1.19, 1.89)	*1.48 (1.24, 1.76)	1.35 (0.99, 1.85)
Model 4	*1.39 (1.17, 1.67)	0.92 (0.75, 1.12)	*1.25 (1.03, 1.52)	1.09 (0.77, 1.55)
Model 5	1.11 (0.92, 1.33)	0.99 (0.75, 1.32)	1.04 (0.85, 1.27)	0.88 (0.61, 1.26)
TyG<br>Model 1	*2.02 (1.77, 2.29)	*1.84 (1.53, 2.22)	*1.72 (1.51, 1.96)	*1.55 (1.24, 1.93)
Model 2	*1.91 (1.67, 2.18)	*1.81 (1.49, 2.19)	*1.62 (1.42, 1.86)	*1.53 (1.23, 1.92)
Model 3	*1.84 (1.61, 2.10)	*1.80 (1.49, 2.18)	*1.59 (1.38, 1.83)	*1.51 (1.20, 1.89)
Model 4	*1.79 (1.52, 2.10)	*1.67 (1.32, 2.12)	*1.47 (1.26, 1.72)	*1.36 (1.05, 1.76)
Model 5	NA	NA	NA	NA

Model 1: Adjusted for age and cohort

Model 2: Additionally adjusted for BMI

Model 3: Additionally adjusted for SBP, treatment for hypertension, smoking, prevalent CVD

Model 4: Additionally adjusted for HDL, triglycerides, serum lipid reducing agents

Model 5: Additionally adjusted for fasting glucose; Hazard ratios are presented per 1 standard deviation increase in the marker. [e]Marker is naturally log-transformed; *$p<0.05$; NA, Not Applicable

ESM Table 3. Baseline characteristics of study participants with DXA measurements on body fat (N = 3028).

Characteristic	Women (1770)	Men (1258)	p-value
Age (years)	63.4 ± 5.9	63.3 ± 5.8	0.3
Systolic Blood pressure (mm/Hg)	148.8 ± 21.6	147.1 ± 20.3	0.022
Treatment for hypertension, n (%)	463 (36.9)	269 (30.1)	0.001
Prevalent cardiovascular disease, n	146 (8.2)	255 (20.3)	< 0.001
Serum lipid reducing agents use, n	357 (20.2)	288 (22.9)	0.06
Current smokers, n (%)	234 (13.2)	167 (13.3)	< 0.001
Total cholesterol (mmol/l)	5.9 ± 0.9	5.4 ± 0.9	< 0.001
*Glucose (mmol/l)	5.4 (4.7 – 6.6)	5.5 (4.7 – 6.7)	0.001
BMI (kg/m^2)	27.5 ± 4.4	26.9 ± 3.2	0.003
Waist circumference (cm)	88.3 ± 10.8	98.3 ± 9.9	< 0.001
*High density lipoprotein cholesterol	1.5 (1.0 – 2.3)	1.3 (0.8 – 1.9)	< 0.001
*Triglycerides (moml/l)	1.3 (0.7 – 2.7)	1.3 (0.7 – 2.9)	0.2
DXA measurements	Women (1770)	Men (1258)	
*Android fat %	3.3 (1.8 – 4.5)	3.1 (1.6 – 4.3)	< 0.001
*Gynoid fat %	6.3 (4.5 – 8.1)	3.9 (2.6 – 5.3)	< 0.001
*Android/Gynoid	0.5 (0.3 – 0.7)	0.8 (0.5 – 1.1)	< 0.001
*Total fat mass %	39.3 (27.2 – 48.6)	27.6 (16.9 – 37.1)	< 0.001

Values are presented as means ± standard deviation or * median (inter-quartile range). Abbreviations: BMI, body mass index. Triglycerides were not available in RSI-4 and RSII-2 and were taken from RSI-3 (the closest previous visit).

Baseline characteristics of participants with DXA differ significantly (p < 0.001) in their baseline characteristics from participants without available DXA measures, whereas they do not have significant difference for prevalent CVD (P=0.3); HDL cholesterol (p= 0.055) and triglycerides (p = 0.7). However, given that is the same cohort of the Rotterdam Study, but different visits, the subjects with DXA included in the analyses, are the subset of the study sample without DXA measures, which survived until the next visit of the Rotterdam Study, where DXA was measured.

ESM Table 4. Associations between DXA measurements on body fat and incident type 2 diabetes in obese/non-obese subjects (N= 3028).

DXA measurements	Incident type 2 diabetes			
	Women		Men	
	BMI < 30<br>(N= 114)	BMI ≥ 30<br>(N= 70)	BMI < 30<br>(N= 105)	BMI ≥ 30<br>(N= 30)
	HR(95%CI)	HR(95%CI)	HR(95%CI)	HR(95%CI)
[e]Android fat mass %<br>Model 1	*1.80 (1.35, 2.41)	1.38 (0.78, 2.43)	*1.58 (1.19, 2.09)	0.87 (0.36, 2.14)
Model 2	*1.54 (1.09, 2.19)	1.04 (0.57, 1.89)	*1.39 (1.004, 1.94)	1.09 (0.41, 2.93)
Model 3	*1.49 (1.05, 2.11)	0.98 (0.53, 1.84)	1.39 (0.99, 1.93)	1.05 (0.37, 2.95)
Model 4	1.38 (0.96, 1.98)	0.78 (0.42, 1.48)	1.32 (0.93, 1.88)	0.94 (0.32, 2.78)
Model 5	1.34 (0.95, 1.89)	0.62 (0.32, 1.21)	1.30 (0.91, 1.85)	1.14 (0.36, 3.66)
[e]Gynoid fat mass %<br>Model 1	0.82 (0.59, 1.13)	0.89 (0.45, 1.77)	1.23 (0.88, 1.70)	0.99 (0.41, 2.42)
Model 2	*0.50 (0.34, 0.74)	0.61 (0.30, 1.25)	0.98 (0.68, 1.41)	1.25 (0.47, 3.29)
Model 3	*0.52 (0.35, 0.76)	0.64 (0.32, 1.29)	0.98 (0.68, 1.41)	1.25 (0.46, 3.42)
Model 4	*0.57 (0.38, 0.84)	0.77 (0.36, 1.64)	1.09 (0.74, 1.60)	1.06 (0.39, 2.93)
Model 5	0.77 (0.51, 1.15)	0.74 (0.34, 1.61)	1.05 (0.71, 1.55)	1.07 (0.38, 3.04)
[e]Android/Gynoid<br>Model 1	*2.08 (1.58, 2.74)	1.34 (0.83, 2.16)	*1.64 (1.19, 2.26)	0.91 (0.35, 2.36)
Model 2	*1.92 (1.44, 2.57)	1.28 (0.78, 2.08)	*1.49 (1.07, 2.09)	0.92 (0.35, 2.38)
Model 3	*1.89 (1.41, 2.52)	1.24 (0.75, 2.04)	1.10 (0.72, 1.69)	2.09 (0.58, 7.54)
Model 4	*1.77 (1.29, 2.41)	0.97 (0.56, 1.66)	1.01 (0.64, 1.59)	2.9 (0.68, 6.7)
Model 5	*1.51 (1.09, 2.08)	0.85 (0.48, 1.50)	1.32 (0.90, 1.92)	1.06 (0.35, 3.22)
[e]Total fat mass %<br>Model 1	1.39 (0.96, 1.99)	0.87 (0.33, 2.31)	*1.56 (1.14, 2.14)	1.17 (0.42, 3.23)
Model 2	0.70 (0.43, 1.13)	0.34 (0.11, 1.00)	1.30 (0.89, 1.91)	1.71 (0.54, 5.42)
Model 3	0.71 (0.45, 1.12)	0.36 (0.13, 1.01)	1.29 (0.88, 1.90)	1.68 (0.55, 5.43)
Model 4	0.74 (0.46, 1.20)	0.39 (0.13, 1.15)	1.38 (0.91, 2.09)	1.39 (0.40, 4.79
Model 5	1.01 (0.61, 1.64)	0.42 (0.13, 1.35)	1.37 (0.91, 2.06)	1.65 (0.46, 5.96)

Model 1: Adjusted for age and cohort

Model 2: Additionally adjusted for BMI

Model 3: Additionally adjusted for SBP, treatment for hypertension, smoking, prevalent CVD

Model 4: Additionally adjusted for HDL, triglycerides, serum lipid reducing agents

Model 5: Additionally adjusted for fasting glucose; Hazard ratios are presented per 1 standard deviation increase in the marker. [e]Marker is naturally log-transformed; *$p<0.05$
